# Parenteral hydration and the cognitively impaired: the important role of subcutaneous hydration

**DOI:** 10.1007/s41999-026-01513-y

**Published:** 2026-05-30

**Authors:** Mathias Brix Danielsen, Stig Andersen, Shaun O’Keeffe

**Affiliations:** 1 Department of Geriatric Medicine, Aalborg University Hospital, Aalborg, Denmark; 2 Department of Clinical Medicine, Aalborg Universitet, Aalborg, Denmark; 3 Department of Medicine, North Denmark Regional Hospital, Hjørring, Denmark; 4 Department of Geriatric Medicine, Galway University Hospitals, Galway, Ireland

**Keywords:** Delirium, Cognitive impairment, Subcutaneous hydration, Hypodermoclysis, Fluid therapy, Agitation

## Abstract

**Purpose:**

Dehydration is common among those admitted to hospital with delirium or dementia and may itself contribute to cognitive impairment. Agitated behaviors are also common in such patients and may lead to difficulty in maintenance of peripheral IV cannulas. In this paper, we examine the potential advantages and drawbacks of subcutaneous (SC) compared with intravenous (IV) hydration in patients with cognitive impairment and mild-to-moderate dehydration.

**Methods:**

We reviewed and summarized the available literature on SC versus IV hydration in patients with cognitive impairment and the impact on agitation and cannula maintenance in particular.

**Results:**

Compared with IV hydration, SC hydration is faster to establish and associated with fewer adverse effects. A meta-analysis of randomized controlled trials found that SC hydration in patients with cognitive impairment results in a lower risk of agitation (relative risk 0.40 [95% CI 0.22–0.72]) compared with IV hydration. Furthermore, there is an 80% reduced risk of catheter dislodgment. It is likely that the reduced risk of agitation and catheter dislodgment is causally related to the fact that subcutaneous access is less traumatic approach for a population at high risk for agitation.

**Conclusion:**

SC hydration has many advantages and few disadvantages for those with mild-to-moderate dehydration and cognitive impairment who need parenteral hydration. It should, in our opinion, always be considered in this population. It is important, however, that ease of use does not lead to overuse, especially where low-intake dehydration may represent part of the natural course of advanced illness.

## Introduction

A third of all patients admitted to general hospitals for acute care have delirium or dementia or both [1, 2]. Both delirium and dementia are associated with excess mortality and poorer functional outcome. Dementia is the main risk factor for delirium; conversely, delirium has been linked with worsening cognitive decline and incident dementia [2, 3]. Both are also frequently associated with agitated behavior and motor hyperactivity causing patient distress and difficulties for healthcare staff [3, 4].

Current and impending dehydration is present in between a third and half of older adults admitted to acute hospital and is associated with increased short and long team mortality [5, 6]. The relationship between cognitive impairment and dehydration is bidirectional and complex. Cognitive difficulties contribute to the risk of dehydration, and dehydration is common among those admitted to hospital with delirium or dementia [7]. Conversely, low-intake dehydration generally is a predictor of impaired cognition and is both a predisposing and precipitating factor for delirium [8, 9].

## Intravenous versus subcutaneous hydration

In older adults with dehydration due to reduced oral intake, the preferred initial rehydration strategy should always be encouragement and support of oral fluids. However, when parenteral hydration is required, as it inevitably will be in some, intravenous (IV) access is traditionally preferred in an acute hospital setting. This is always the appropriate choice when there is severe dehydration or volume depletion, characterized by hemodynamic instability, significant electrolyte disturbance, or the need for rapid fluid resuscitation [10].

However, subcutaneous (SC) hydration (hypodermoclysis) is now recognized, including in the recent European Society for Clinical Nutrition and Metabolism (ESPEN) guidelines [11], as a safe and effective alternative for older people with mild-to-moderate dehydration or who need supplemental fluid due to insufficient oral intake. The mild-to-moderate dehydration in older adults can be defined by reduced oral intake combined with laboratory indicators of dehydration such as elevated serum urea-to-creatinine ratio, hypernatremia, or increased serum osmolality (> 300 mOsm/kg) [12].

With SC hydration, fluid delivered into the subcutaneous space is absorbed into the capillaries through passive diffusion. Radioisotope studies have confirmed that the fluid enters the circulation and suggest that, even in frail, unwell older patients with reduced albumin levels, 1 L of isotonic fluid can be administered over 8 h, which is consistent with standard recommendations [13, 14].

In this paper, we argue that there are compelling reasons and evidence to support why SC hydration must be considered when parenteral hydration is clinically indicated for those with cognitive impairment who have mild-to-moderate dehydration and/or reduced oral intake.

## Peripheral IV cannulas and the patient with cognitive impairment

Delirium is a multifactorial syndrome in older people, often resulting from the interaction of predisposing vulnerability factors, including cognitive impairment, with precipitating (hospitalization-related) factors including procedures and immobilizing devices or restraints. In a landmark study, Inouye and colleagues showed that a multicomponent intervention, including encouragement of oral intake of fluids in those with poor intake, reduced delirium incidence by 40% [15].

It is possible that insertion of IV cannulas, and the measures subsequently required to protect the catheter also play a role in the development or worsening of delirium. Nearly half of all first IV insertion attempts fail, and many older people require multiple attempts for successful insertion, causing pain and anxiety, especially in those with cognitive impairment [16]. Clinicians suggest that patient distress, sometimes manifesting as aggression, is common during invasive procedures such as phlebotomy and insertion of IV cannulas; although this can be mitigated by communication strategies, restraint is sometimes used, or some procedures may be abandoned [17]. In a large, prospective observational study of medical inpatients, “medical restraints”, such as intravenous or oxygen tubing, were an independent predictor of the severity of delirium symptom scores [18].

Maintenance of peripheral IV cannulas after insertion is also a problem, and the overall failure rate is between 35 and 50% [19]. There are many reasons for such failures, but dislodgement is one of the most common factors accounting for up to half of failures [20]. This mainly occurs when people are receiving fluids (rather than when the catheter is capped and only used for injecting, for example, antibiotics). Each IV cannula replacement again results in discomfort and distress for the patient, with consequences including bleeding and skin tearing [21]. Essential treatment may be delayed. Also, several authors have commented on the very significant costs for health services associated with IV cannula failure (compounded by the enormous costs of treating cannula-related bloodstream infection) [19, 22].

By far the most important factor contributing to the dislodgement of IV cannulas is cognitive impairment [21, 23]. A survey of 1561 healthcare professionals identified the “confused patient” as accounting for 80% of dislodgements, usually by physically removing the device [21]. Another study identified agitation, delirium, and dementia as important contributors to the unplanned removal of cannulas and other devices and noted that most such incidents occurred at night when behavioral manifestations of cognitive impairment tend to be worse [23]. A high rate of dislodgement of peripheral cannulas in COVID-19 was also attributed to the high rates of delirium in the population [24].

Better IV cannula securement techniques can address some dislodgement cases but will have little impact when a cognitively impaired patient is intent on physical removal of a cannula. Instead, unfortunately, a common response remains administration of psychotropic medication or physical restraint [17, 23], both of which will potentially worsen agitation and distress and lead to worse outcomes for the patient.

## The potential benefits of subcutaneous hydration in cognitive impairment

Compared with IV access, SC access is simpler, requires less technical skill, less patient cooperation, and involves less physical trauma. Some significant complications associated with venous access, such as phlebitis and catheter-related bloodstream infections (occurring in 16–23% and 0–2.2% IV cannulas respectively in prospective studies [19]), cannot occur with SC infusions. An IV cannula inserted in the arm is clearly visible to the patients and is perhaps an annoyance if interfering with movement or with eating and drinking. In contrast, a SC cannula can be placed in the abdomen or back where it is less bothersome and, for someone with cognitive impairment, may be “out of sight and out of mind”. Infusion rates are slower with the SC route, which highlights the need for careful selection of suitable patients; on the other hand, fluids cannot accidentally be given too rapidly as can occur with IVs.

A recent meta-analysis of randomized controlled trials (RCTs) of SC and IV hydration and a new RCT on this topic confirm the benefits of SC hydration [25, 26]. Overall, SC access is significantly faster to achieve [26, 27], and adverse effects are markedly and significantly less common in patients treated with SC hydration than in those treated with IV hydration with a relative risk of 0.62 (95% CI 0.53–0.71).

**Fig. 1:**
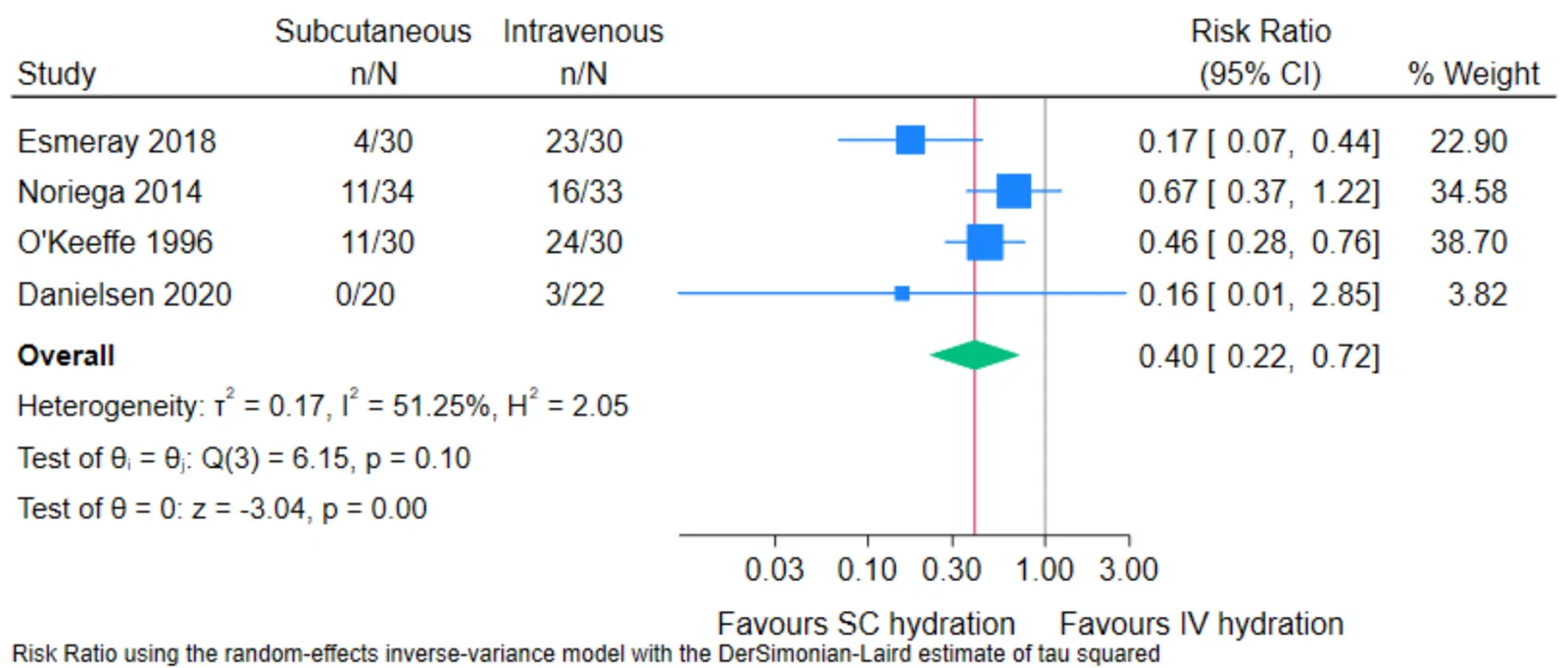
Adjusted meta-analysis with inclusion of the new RCT on the risk of agitation or delirium comparing subcutaneous with intravenous hydration [25, 26, 28–30]. Reproduced with permission from Danielsen 2021 [31]

Two outcomes are of particular relevance for older patients with, or at risk for, cognitive impairment. Agitation occurred in 26/114 receiving SC and 66/115 receiving IV hydration [Relative risk (RR) 0.40, CI 0.22–0.72, Table 1, Fig. 1]. Cannula dislodgment occurred in 3/743 SC infusions and 16/740 receiving IV hydration [RR 0.19 (95% confidence interval (CI) 0.05–0.64)]. However, the assessment of agitation varied across studies, and only one study had blinded outcome assessors (see Table 1). Regardless, these are clearly clinically meaningful results, even accepting the wide confidence limits and the flaws in individual studies [25].

**Table 1:** Description of trials evaluating the risk of delirium or agitation comparing subcutaneous and intravenous hydration

Study	Study design (*n*)	Participants including cognitive level	Intervention Inc. amount and type	Method of evaluation change in cognitive function	Result of cognitive function
O’Keeffe [28]	RCT *n* = 30 in IV *n* = 30 in SC	Mean age: 82.5 ± 6.5 years. All cognitive impaired	Average of 1.4 L/day of either 0.9% saline or 0.45% saline in 5% glucose	Nursing staff noted any agitation or disturbance related to the infusion	Experienced agitation: IV: 24/30 SC: 11/30
Noriega [29]	RCT *n* = 33 in IV *n* = 34 in SC	Mean age: 85.4 ± 7.5 years. 23 in IV and 28 in SC group with cognitive reduction or dementia	Average of 1400 mL/day. Most received 0.9% Saline, followed by 5% dextrose	The presence of psychomotor agitation was documented by regular monitoring of daily nursing and clinical course of registration of physical and/or pharmacological restraint	Experienced agitation: IV: 16/33 SC: 11/34
Esmeray [30]	Cross-over RCT *n* = 30	Mean age: 82.0 ± 8.8 years. All with a diagnoses of dementia	1000 mL of 0.9% Saline	Clinical evaluation of agitation that develop during or after the infusion	Experienced agitation: IV: 23/30 SC: 4/30
Danielsen [26]	RCT *n* = 22 in IV *n* = 20 in SC	Mean age: 82 ± 7.2 years. Excluded if cognitive reduced	1000 mL of 0.9% saline	Evaluated using Confusion Assessment Method	Positive CAM: IV 3/22 SC 0/20

*n* number of participants, *RCT* randomized controlled trial, *IV* intravenous, *SC* subcutaneous, *CAM* Confusion Assessment Method

It seems most likely that the more than halving of the risk of agitation and the 80% reduction in dislodgement with SC hydration are causally linked, although only one paper explicitly noted that agitation was related to the cannula or drip [28]. It remains unclear whether the reduction in ‘agitation’—often poorly defined in studies—represented avoidance of behavioral consequences of dementia and delirium or a prevention of delirium. The only RCT examining the issue found delirium in 0/20 (0%) patients receiving SC and 3/22 (14%) receiving IV hydration (*p* = 0.23) but was underpowered to find a significant difference (recruitment being restricted by the advent of the COVID-19 pandemic) [26]. Regardless whether SC hydration reduces the incidence of delirium, the large reduction in agitation seems clinically relevant. There is a need for future trials with a primary focus on the risk of delirium comparing SC and IV hydration.

Although only one study formally to date has explicitly reported on the reduced costs associated with use of SC versus IV hydration, it is likely that more use of the SC approach will lead to significant savings given that the butterfly cannulas used are less expensive than IV cannulas and are less likely to be dislodged, and because there is no potential for cannula-related bloodstream infections [28].

Finally, although most studies to date have been in hospitalized patients, the greatest potential impact on reducing agitation might be through admission avoidance in nursing home residents with better management of impending dehydration using SC fluids; this warrants further study [11].

## The potential drawbacks of subcutaneous hydration in cognitive impairment

One significant limitation of SC hydration is that the maximum recommended infusion rate is typically 1–1.5 L/day, limiting its use in patients requiring rapid fluid resuscitation or large volumes. Local complications such as edema, erythema, or discomfort may occur, although these are usually mild and self-limiting [25]. Electrolyte disturbances may occur if inappropriate fluids are used, and the correction of severe electrolyte disturbances using SC hydration has not been studied. In patients with generalized edema and/or very low serum albumin, SC hydration is contraindicated due to concerns about poor absorption [10, 32].

Perhaps the greatest potential drawback of SC hydration is that ease of use will result in overuse. Artificial hydration should be considered only when oral intake is temporarily insufficient and potentially reversible, such as during acute illness, infection, medication effects, or delirium impairing oral intake. In contrast, in patients with advanced dementia who have permanently lost the ability to swallow safely, the benefits of artificial hydration are uncertain and must be weighed against the goals of care. In such circumstances, low-intake dehydration may represent part of the natural course of advanced illness, and a comfort-focused approach without parenteral hydration is often the appropriate strategy [33, 34].

## Conclusion and recommendation

The choice between SC and IV hydration should always be tailored to the individual patient’s needs and circumstances. For those with cognitive impairment, SC hydration has many advantages and few disadvantages. It is intuitively a kinder, less traumatic approach for a population at high risk for distress and agitation related to hospitalization. In our opinion, the evidence, while imperfect, might be “good enough” to warrant a recommendation that SC hydration should be considered in patients where parenteral hydration is indicated and who have mild-to-moderate dehydration or need supplemental parenteral fluid due to poor oral intake.

## Data Availability

No unpublished data were generated or analyzed. All referenced data is avalable in the cited papers.

## References

[1] Goldberg SE, Whittamore KH, Harwood RH, Bradshaw LE, Gladman JR, Jones RG (2012) The prevalence of mental health problems among older adults admitted as an emergency to a general hospital. Age Ageing 41:80–86. 10.1093/ageing/afr106 21890483 PMC3234074

[2] Gibb K, Seeley A, Quinn T, Siddiqi N, Shenkin S, Rockwood K et al (2020) The consistent burden in published estimates of delirium occurrence in medical inpatients over four decades: a systematic review and meta-analysis study. Age Ageing 49(3):352–360. 10.1093/ageing/afaa040 32239173 PMC7187871

[3] Fong TG, Inouye SK (2022) The inter-relationship between delirium and dementia: the importance of delirium prevention. Nat Rev Neurol 18(10):579–596. 10.1038/s41582-022-00698-7 36028563 PMC9415264

[4] Sourial R, McCusker J, Cole M, Abrahamowicz M (2001) Agitation in demented patients in an acute care hospital: prevalence, disruptiveness, and staff burden. Int Psychogeriatr 13(2):183–197. 10.1017/s1041610201007578 11495393

[5] Sanson G, Marzinotto I, De Matteis D, Boscutti G, Barazzoni R, Zanetti M (2020) Impaired hydration status in acutely admitted older patients: prevalence and impact on mortality. Age Ageing. 10.1093/ageing/afaa264 33320928

[6] Li S, Xiao X, Zhang X (2023) Hydration status in older adults: current knowledge and future challenges. Nutrients. 10.3390/nu15112609 37299572 PMC10255140

[7] George J, Rockwood K (2004) Dehydration and delirium-not a simple relationship. J Gerontol A Biol Sci Med Sci 59(8):811–812. 10.1093/gerona/59.8.m811 15345730

[8] Glover A, Bradshaw LE, Watson N, Laithwaite E, Goldberg SE, Whittamore KH et al (2014) Diagnoses, problems and healthcare interventions amongst older people with an unscheduled hospital admission who have concurrent mental health problems: a prevalence study. BMC Geriatr. 10.1186/1471-2318-14-43 24694034 PMC3992161

[9] Barani N, Jackson AC, Sharifi F, Bahramnezhad F (2025) The relationship between thirst and the incidence of delirium in patients admitted to intensive care units: a cross sectional study. Health Sci Rep 8(12):e71485. 10.1002/hsr2.71485 41324094 PMC12660157

[10] Arcani R, George J, Correard F, Bérard C, Villani P, Daumas A (2025) [Subcutaneous infusion: indications, practical considerations, and tolerability]. Rev Med Interne 47(2):76–83. 10.1016/j.revmed.2025.11.002 41412857

[11] Volkert D, Beck AM, Cederholm T, Cruz-Jentoft A, Hooper L, Kiesswetter E et al (2022) ESPEN practical guideline: clinical nutrition and hydration in geriatrics. Clin Nutr 41(4):958–989. 10.1016/j.clnu.2022.01.024 35306388

[12] Beck AM, Volkert D (2023) Proper nutrition and hydration are human rights: also and especially for older patients. Eur Geriatr Med 14(3):407–410. 10.1007/s41999-023-00771-4 37000400 PMC10261183

[13] Danielsen MB, Jødal L, Riis J, Karmisholt JS, Valdórsson Ó, Jørgensen MG et al (2022) Absorption rate of subcutaneously infused fluid in ill multimorbid older patients. PLoS ONE 17(10):e0275783. 10.1371/journal.pone.0275783 36215232 PMC9550057

[14] Lipschitz S, Campbell AJ, Roberts MS, Wanwimolruk S, McQueen EG, McQueen M et al (1991) Subcutaneous fluid administration in elderly subjects: validation of an under-used technique. J Am Geriatr Soc 39(1):6–9. 10.1111/j.1532-5415.1991.tb05898.x 1846157

[15] Inouye SK, Bogardus ST, Charpentier PA, Leo-Summers L, Acampora D, Holford TR et al (1999) A multicomponent intervention to prevent delirium in hospitalized older patients. N Engl J Med 340(9):669–676. 10.1056/NEJM199903043400901 10053175

[16] Ní Chróinín D, Ray-Barruel G, Carr PJ, Frost SA, Rickard CM, Mifflin N et al (2023) The burden of peripheral intravenous catheters in older hospital inpatients: a national cross-sectional study part of the one million global peripheral intravenous catheters collaboration. Australas J Ageing 42(1):98–107. 10.1111/ajag.13068 35384222

[17] Fox A, Johnston S, Patterson S WK, Bail K, Hutt L et al (2025) Perceptions and practices of clinicians undertaking invasive procedures with patients experiencing delirium in hospital: a sequential mixed methods study. Nurs Health Sci 27(2):e70154. 10.1111/nhs.70154 40442059 PMC12122409

[18] McCusker J, Cole M, Han L AM, Podoba JE, Ramman‐Haddad L (2001) Environmental risk factors for delirium in hospitalized older people. J Am Geriatr Soc 49(10):1327–1334. 10.1046/j.1532-5415.2001.49260.x 11890491

[19] Helm RE, Klausner JD, Flint LM KJD, Huang E (2019) Accepted but unacceptable: peripheral IV catheter failure. J Infus Nurs 42:151–164. 10.1097/NAN.0000000000000326 30985565

[20] Jackson A (2012) Retrospective comparative audit of two peripheral IV securement dressings. Br J Nurs 21:S10–S15. 10.12968/bjon.2012.21.Sup1.S10

[21] Moureau N (2018) Impact and safety associated with accidental dislodgement of vascular access devices: a survey of professions, settings, and devices. J Assoc Vasc Access 23(4):203–215. 10.1016/j.java.2018.07.002

[22] van Loon FH, Leggett T, Bouwman AR, Dierick-van Daele AT (2020) Cost-utilization of peripheral intravenous cannulation in hospitalized adults: an observational study. J Vasc Access 21(5):687–693. 10.1177/1129729820901 31969049

[23] Kato M, Fauziah W, Yamashita T, Nishijima S, Kima M, Iida M et al (2012) Prevalence and prevention of unplanned removal of tubes and catheters among hospitalized patients. Int J Caring Sci 14(1):385

[24] Gidaro A, Vailati D, Lugli F GM, Casella F, Cogliati C et al (2022) Retrospective survey from vascular access team Lombardy net in COVID-19 era. J Vasc Access 23(4):532–537. 10.1177/1129729821997252 33618564

[25] Danielsen MB, Andersen S, Jorgensen MG WE (2020) Harms and benefits of subcutaneous hydration in older patients: systematic review and meta‐analysis. J Am Geriatr Soc 68(12):2937–2946. 10.1111/jgs.16707 33411351

[26] Danielsen MB, Worthington E, Møller JM KJS, Jørgensen MG, Andersen S (2022) Adverse effects of subcutaneous vs intravenous hydration in older adults: an assessor-blinded randomised controlled trial (RCT). Age Ageing. 10.1093/ageing/afab193 34651171

[27] Özkaya H, Baktır Altuntaş S, Öz Esmeray G, Baki ŞE, Beşel A (2025) Hypodermoclysis versus intravenous hydration in palliative medicine: clinical effectiveness. BMJ Support Palliat Care. 10.1136/spcare-2025-005882. 41314778

[28] O’Keeffe ST, Lavan JN (1996) Subcutaneous fluids in elderly hospital patients with cognitive impairment. Gerontology 42(1):36–39. 10.1159/000213768 8641599

[29] Noriega OD, Blasco SA (2014) Eficacia de la vía subcutánea frente a la hidratación intravenosa en el paciente anciano hospitalizado: estudio controlado aleatorizado. Rev Esp Geriatr Gerontol 49(3):103–107. 10.1016/j.regg.2013.12.003 24484688

[30] Esmeray G, Şenturan L, Döventaş A (2018) A study on efficacy of hydration administered by subcutaneous infusion in geriatric patients. Turk J Geriatr 21(3):438–445. 10.31086/tjgeri.2018344059

[31] Danielsen MAB (2021) Subcutaneous hydration in geriatric patients. Aalborg Universitet. Det Sundhedsvidenskabelige Fakultet. Ph.D.-Serien. Aalborg University. https://vbn.aau.dk/da/publications/subcutaneous-hydration-in-geriatric-patients

[32] Broadhurst D, Cooke M, Sriram D, Barber L, Caccialanza R, Danielsen MB (2023) International consensus recommendation guidelines for subcutaneous infusions of hydration and medication in adults: an e-Delphi consensus study. J Infus Nurs. 10.1097/NAN.0000000000000511 37406334 PMC10306332

[33] Volkert D, Beck AM, Faxén-Irving G, Frühwald T, Hooper L, Keller H et al (2024) ESPEN guideline on nutrition and hydration in dementia—update 2024. Clin Nutr 43(6):1599–1626. 10.1016/j.clnu.2024.04.039 38772068

[34] Ramalho AR, Rocha MJ, Magalhães JA, Santos N, Santana I, Veríssimo MT et al (2025) Feeding-related hospitalizations and outcomes in advanced dementia. Eur Geriatr Med 28:1–9. 10.1007/s41999-025-01366-x PMC13109124 41604097

